# Supplementary material for: Hyperglycaemia induces metabolic dysfunction and glycogen accumulation in pancreatic β-cells
Source: Nat Commun. 2016 Nov 24;7:13496. doi: 10.1038/ncomms13496 (PMC5123088; doi:10.1038/ncomms13496)
Supplement: Supplementary Information — Supplementary Figures 1-8 and Supplementary Tables 1-3. [file ncomms13496-s1.pdf]

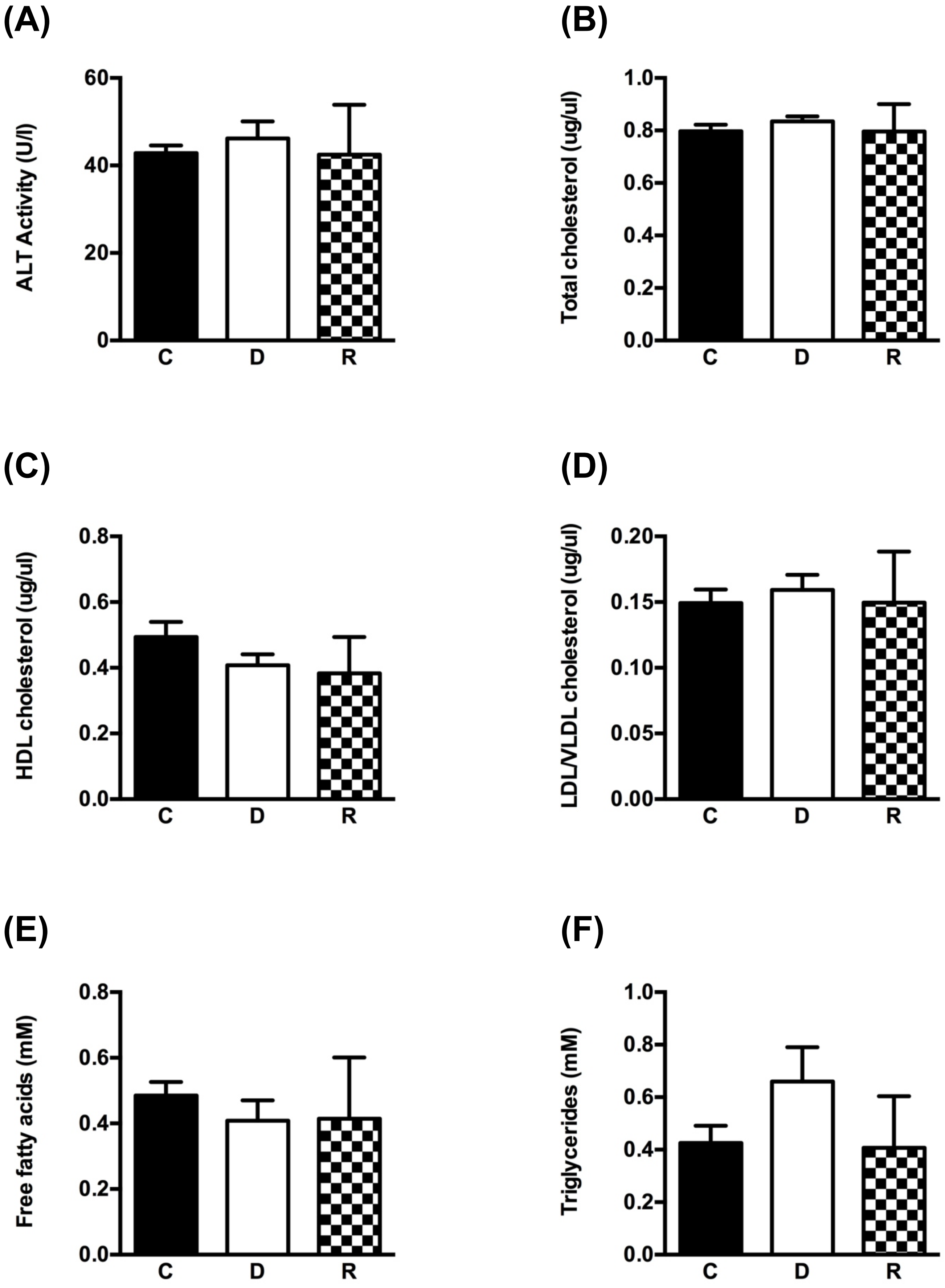

Supplementary Figure 1. Lipid profile in βV59M mice

Alanine aminotransferase (ALT) activity (**A**), total cholesterol (**B**), HDL cholesterol (**C**), LDL/VLDL cholesterol (**D**), free fatty acids (**E**), and triglyceride levels (**F**) in control mice (black bars), βV59M mice 2-4weeks (white bars) after diabetes onset, or after 2-4 weeks of diabetes followed by 2 weeks of glibenclamide therapy (hatched bars). Data are mean±SEM, n=7-20 per genotype.

(A)

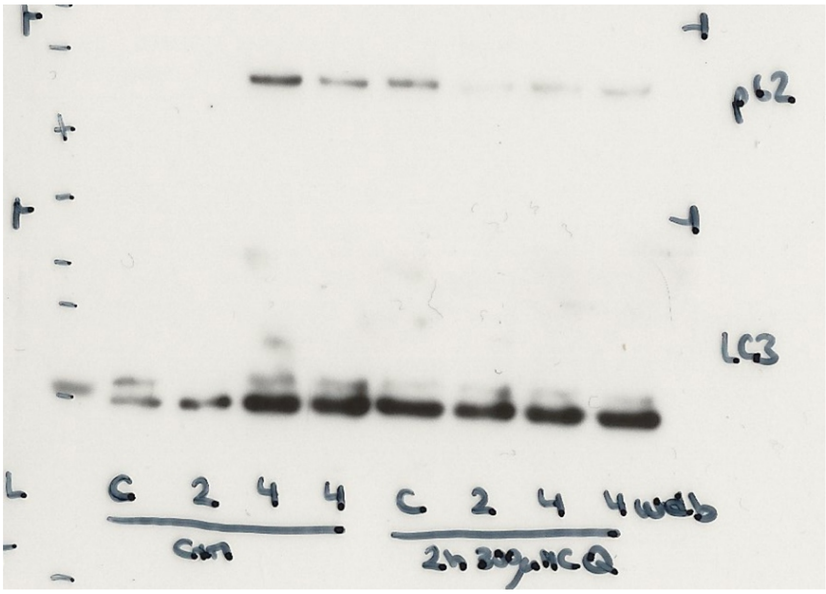

(B)

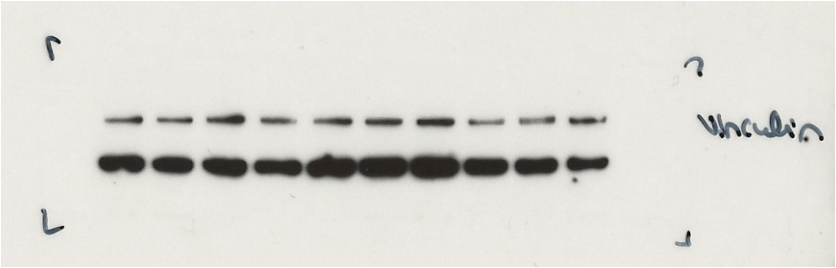

Supplementary Figure 2. Original Western blot images for Figure 5E

(***A***) Western blot image for p62 and LC3B. (***B***) Western blot image for vinculin.

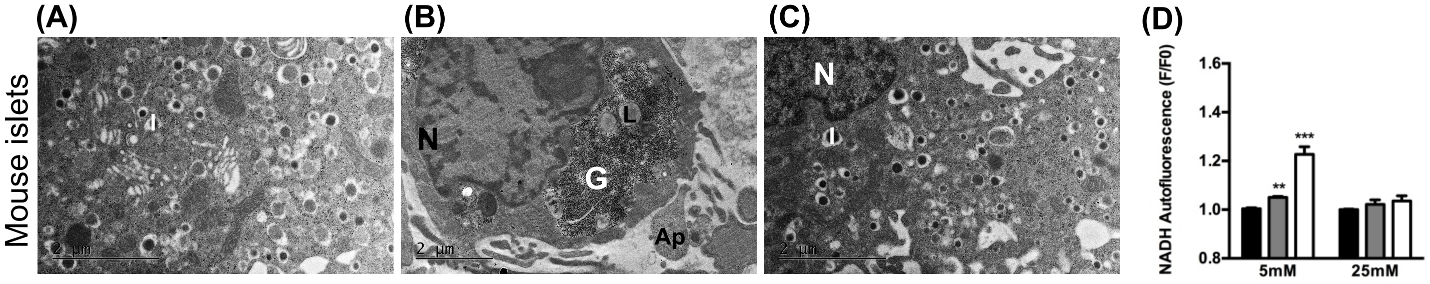

Supplementary Figure 3. Control mouse islets accumulate glycogen *in vitro*

(***A-C***) Representative electron micrographs of isolated islets from control mice following 48 hours culture in 5mM glucose (***A***), 25mM glucose (***B***), or 48h hours in 25mM glucose followed by 24 hours in 5mM glucose (***C***). Data are representative of islets from 3 patients. I, insulin granule. N, nucleus. Ap, apoptotic body. G, glycogen granules. L, lysosome. Scale bars 2µm. (***D***) Mean (±SEM) NAD(P)H autofluorescence produced by 1mM (black bars) 6mM (grey bars) and 20mM glucose (white bars) in islets isolated from control mice and cultured at 5mM glucose or 25mM glucose for 72 hours prior to the experiment (n=4-5).

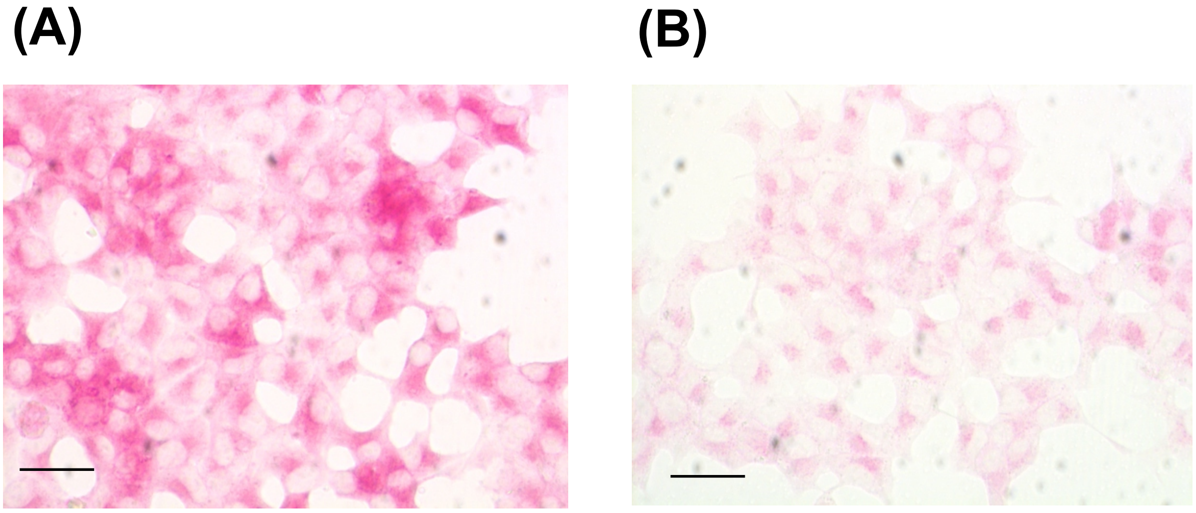

Supplementary Figure 4. Glycogen dissipates from INS-1 cells upon return to low glucose

(***A,B***) Representative images of INS1 cells following 48 hours culture in (***A***) 25mM glucose and (***B***) 48 hours culture in 25mM glucose followed by 24 hours at 5mM glucose. Scale bar, 200μm. Data are representative of >3 separate experiments.

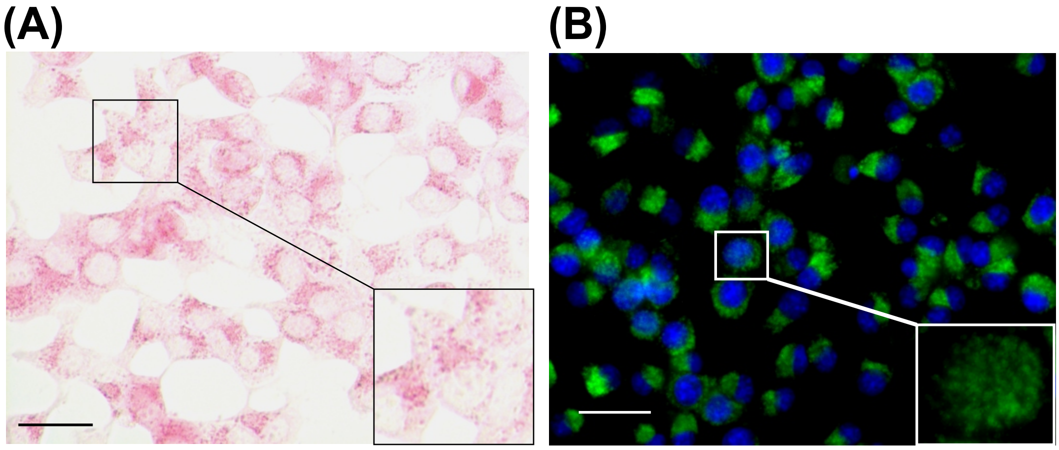

Supplementary Figure 5. Glycogen accumulates in lysosomes in INS-1 cells
(***A,B***) Representative images of INS-1 cells following 48 hours culture in 25mM glucose plus the lysosomal inhibitors pepstatin A (10μg/ml) and E-64d (10μg/ml). (***A***) PAS-staining for glycogen. (***B***) Immunofluorescence staining for the lysosomal marker Lamp-2 (green). Inset shows Lamp-2 positive punctae. Scale bar, 200μm. Data are representative of >3 separate experiments.

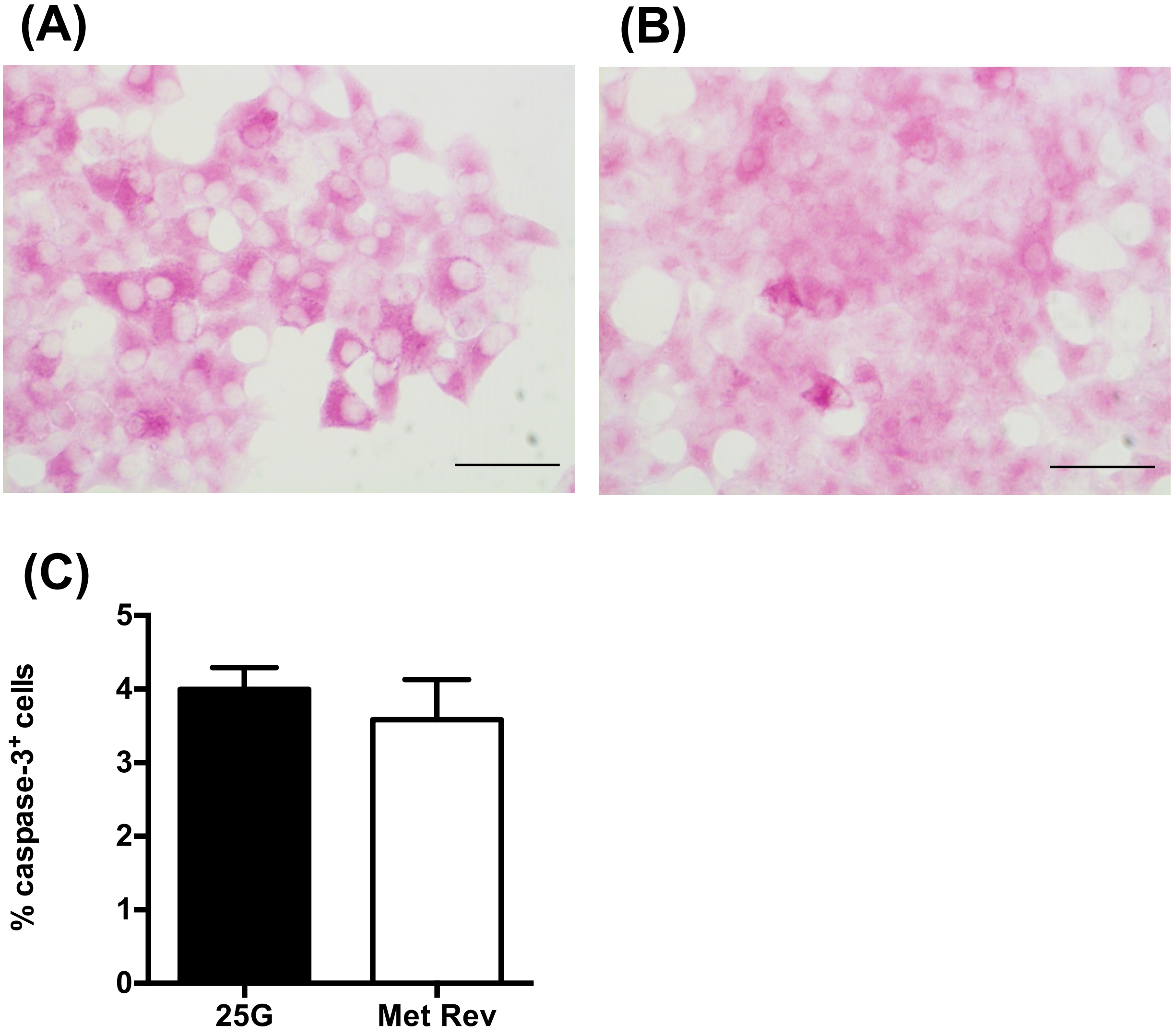

Supplementary Figure 6. Metformin treatment does not reverse glycogen formation

(***A,B***) Representative images of INS-1 cells following 48 hours culture in (***A***) 25mM glucose or (***B***) 48 hours at 25mM glucose then 24-hours with 25mM glucose plus metformin (0.5mM). Scale bar, 200μm. Data are representative of >3 separate experiments. (***C***) Mean±SEM number of cleaved-caspase-3 positive INS-1 cells as a percentage of the total number of INS-1 cells (n=25 fields of view from 3 experiments) in cells cultured for 48 hours in 25mM glucose (black bars), or 48 hours at 25mM glucose then 24-hours with 25mM glucose plus metformin (white bars).

(A)

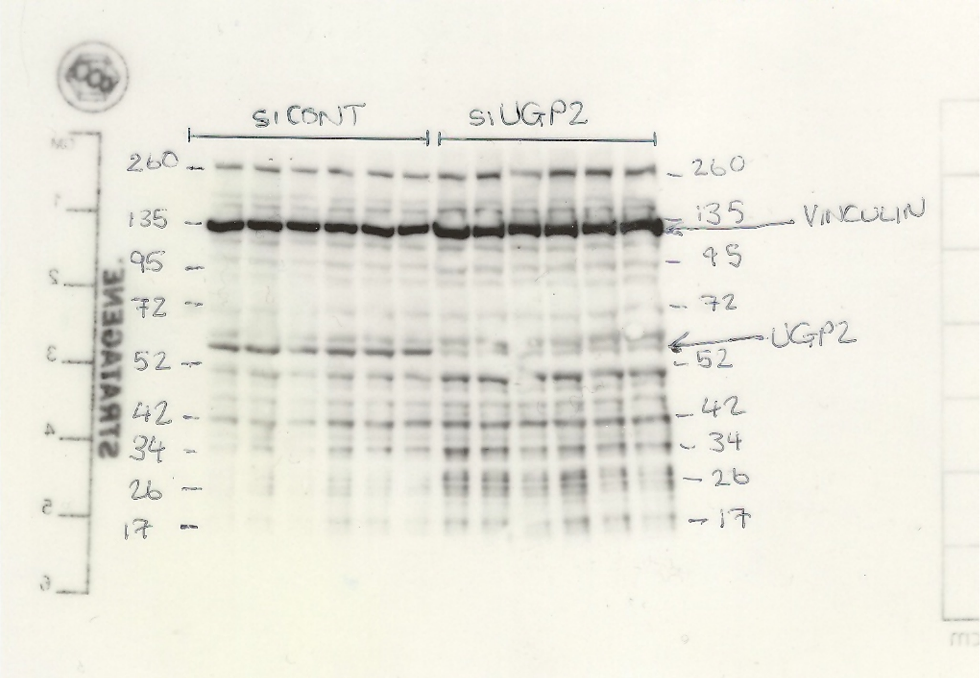

(B)

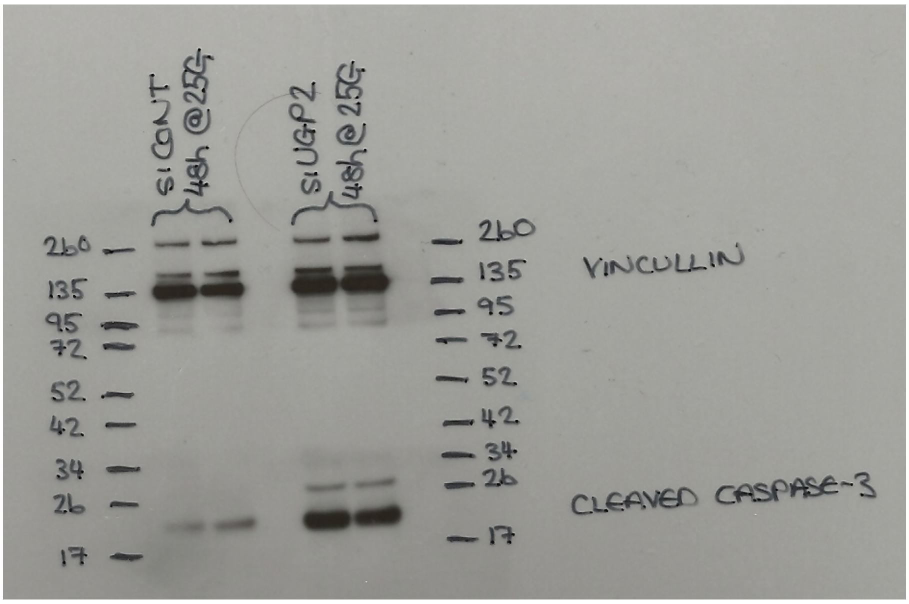

Supplementary Figure 7. Original Western blot images for Figure 7

(***A***) Western blot image for UGP2 and vinculin. (***B***) Western blot image for cleaved caspase-3 and vinculin.

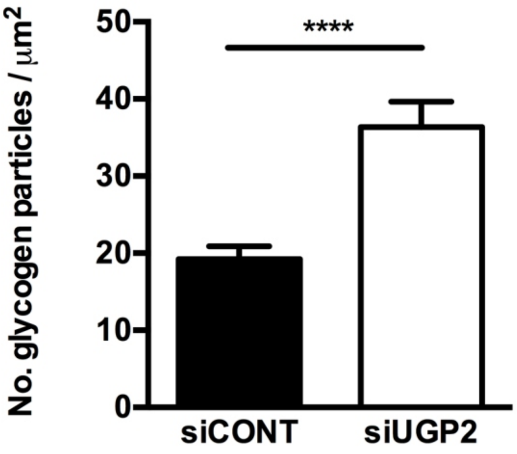

Supplementary Figure 8. Silencing UGP-2 increases glycogen content
Mean (±SEM) number of glycogen particles identified from n=31 cells using electron microscopy in INS-1 cells 72h post UGP2 knockdown that were cultured for 48h at 25mM glucose. siCONTROL (black bar) and siUGP2 (white bar) treated cells. ***$p<0.001$ (Mann-Whitney test).

(A)

Gene Symbol	Gene Name	Control	2wk	Fold change
Aldob	Aldolase B, fructose-bisphosphate	389	5496	14.4
Fbp1	Fructose bisphosphatase 1	986	3851	4.3
Pfkfb3	6-phosphofructo-2-kinase/fructose-2,6-biphosphatase 3	289	1206	4.2
Pdk1	Pyruvate dehydrogenase kinase, isoenzyme 1	770	2770	3.6
Fbp2	Fructose bisphosphatase 2	191	556	2.8
Acaca	Acetyl-Coenzyme A carboxylase alpha	334	929	2.8
Ppp1r3c	Protein phosphatase 1, regulatory (inhibitor) subunit 3C	219	470	2.2
Pgm2l1	Phosphoglucomutase 2-like 1	255	544	2.1
Idh1	Isocitrate dehydrogenase 1 (NADP+), soluble	1475	2995	2.1

(B)

Gene Symbol	Gene Name	Control	2wk	Fold change
Cox6a2	Cytochrome c oxidase, subunit VI a, polypeptide 2	2896	333	6.3
Eno2	Enolase	1471	742	2.0
Fah	Fumarylacetoacetate hydrolase	1345	708	1.9
Pfkfb2	6-phosphofructo-2-kinase/fructose-2,6-biphosphatase 2	2592	1420	1.8
G6pc2	Glucose-6-phosphatase, catalytic, 2	13186	7235	1.8
ATP6	ATP synthase F0 subunit 6 /// predicted gene 10925	2226	1286	1.8
Ogdhl	Oxoglutarate dehydrogenase-like	2293	1328	1.7

Supplementary Table 1. Summary of microarray data

Selected metabolic genes that are significantly up-regulated (**A**) or down-regulated (**B**) in βV59M mice after 2 of weeks of diabetes compared to littermate controls.

Taqman Primers	
**Gene**	**Catalogue number (Applied Biosystems)**
Actb	Mm00607939_s1
ATP6	Mm03649417_g1<br>Rn03296710_s1
Becn1	Mm01265461_m1
Cox6a2	Mm00438295_g1
Fh1	Mm01321349_m1
Fbp1	Mm00490181_m1<br>Rn00561189_m1
Fbp2	Mm00484280_m1
G6pc2	Mm00491176_m1<br>qRnoCIP0033113 (Bio-Rad)
G6pd2	Mm00658204_s1
Gsk3b	Mm00444911_m1<br>Rn01444108_m1
Gys1	Mm01962575_s1<br>Rn01476417_m1
Gys2	Mm0523953_m1<br>Rn00565296_m1
Ogdhl	Mm01221916_m1
Pdk1	Mm00554306_m1
Pfkfb2	Mm00435575_m1
Pgm2l1	Rn01508647_m1
Ppp1r3c	Mm01204084_m1
Pygl	Mm01289790_m1
Pygm	Mm00478582_m1
Ugp2	Rn01434170_m1

Supplementary Table 2: Taqman primer assay ID for the indicated genes.

Antibody	Concentration	Source	Application	Source
Guinea pig anti-insulin	1:500	Made in house	Immunohistochemistry	Made in house
Mouse anti-glucagon	1:500	Sigma (2645)	Immunohistochemistry	Sigma (2645)
Rabbit anti-cleaved caspase-3	1:100, 1:1000	Cell signalling (9664)	Immunohistochemistry, Western blot	Cell signalling (9664)
Rabbit anti-LAMP2	1:200	Sigma (L0668)	Immunohistochemistry	Sigma (L0668)
Mouse anti-ubiquitin	1:100	MBL (Clone IB3)	Immunohistochemistry	MBL (Clone IB3)
Rabbit anti-p62	1:500	MBL (PM045)	Immunohistochemistry, Western Blot, Immuno-gold labelling	MBL (PM045)
Rabbit anti-LC3B	1:1000	Abcam (ab48394)	Western Blot	Abcam (ab48394)
Rabbit anti-vinculin	1:5000	Abcam (ab18058)	Western Blot	Abcam (ab18058)
Rabbit anti-UGP2	1:500	Abcam (ab10627)	Western Blot	Abcam (ab10627)
Anti-guinea pig Texas red	1:50	Vector Laboratories (TI-7000)	Immunohistochemistry	Vector Laboratories (TI-7000)
Anti-mouse Alexa 633	1:500	ThermoFisher (A-21046)	Immunohistochemistry	ThermoFisher (A-21046)
Anti-rabbit Alexa 488	1:200	ThermoFisher (A-11034)	Immunohistochemistry	ThermoFisher (A-11034)
ECL Anti-rabbit IgG, HRP linked	1:2500	GE Healthcare (NA934V)	Western Blot	GE Healthcare (NA934V)

Supplementary Table 3: Antibodies.
